# Validation of the Breast Cancer Screening Beliefs Questionnaire among African Australian women

**DOI:** 10.1186/s12889-016-2793-7

**Published:** 2016-02-04

**Authors:** Cannas Kwok, Olayide Ogunsiji, Chun Fan Lee

**Affiliations:** 1 School of Nursing and Midwifery, Western Sydney University, Sydney, New South Wales Australia; 2 Department of Biostatistics, Singapore Clinical Research Institute, Singapore, Singapore

## Abstract

**Background:**

The Breast Cancer Screening Beliefs Questionnaire (BCSBQ) has been designed as a culturally appropriate instrument for assessing women’s beliefs, knowledge and attitudes to breast cancer and breast cancer screening practices. While it has proved to be a reliable instrument when applied to women of Chinese, Arabic and Korean origin living in Australia, its psychometric properties among women from African backgrounds have not been tested. The aim of this study is to examine the psychometric properties of the BCSBQ among African Australian women.

**Methods:**

The BCSBQ was administered to 284 African Australian women who were recruited from a number of African community organizations and churches. Factor analysis was conducted to study the factor structure. Construct validity was examined using Cuzick’s non-parametric test while Cronbach alpha was used to assess internal consistency reliability.

**Results:**

Exploratory factor analysis results demonstrated that the African-Australian BCSBQ can be conceptualized as a 4-factor model. The third factor, viz. “barriers to mammography”, was split into two separate factors namely, “psychological” and “practical” barriers. The results indicated that the African-Australian BCSBQ had both satisfactory validity and internal consistency. The Cronbach’s alpha of the three subscales ranged between 0.84-0.92. The frequency of breast cancer screening practices (breast awareness, clinical breast-examination and mammography) were significantly associated with attitudes towards general health check-ups and perceived barriers to mammographic screening.

**Conclusions:**

Our study provided evidence to support the psychometric properties of the BCSBQ.in African Australian women. The study moreover demonstrated that the use of the instrument can help health professionals to understand the beliefs, knowledge and attitudes to breast cancer among African Australian women and also the factors that impact on their breast cancer screening practices.

## Background

While Europe and Asia have traditionally been the largest sources of migration to Australia, there also has been large-scale immigration from Africa over the last two decades. As a result, since 2005 people of African origin have become one of the top ten immigrant populations in Australia [1]. The significant increase of culturally diverse population groups has posed a challenge to health care professionals seeking to promote preventive health measures such as breast cancer screening. The task has been made more difficult by the fact that common preventive measures, particularly mammography, are not well promoted in African countries [2].

Yet breast cancer is a health concern for all women regardless of their ethnic background and in fact is the most common form of cancer among first generation female immigrants living in Australia [3]. Although nearly a quarter of the Australian population are overseas-born [1], the proportion of African Australian women diagnosed with breast cancer is currently unknown. And while the five year survival rates among women diagnosed with breast cancer in Australia increased from 72 % to 89 % between 1982 and 2014 [4], this promising statistic does not indicate whether the improvement was common to all ethnic or racial groups. In addition, there is a paucity of evidence on the uptake of breast cancer screening, of mortality and of survival rates among African Australian women.

The bulk of studies on the incidence of breast cancer among immigrant African women emanate from the United States of American (USA) and suggest that the incidence of breast cancer in this group is lower than among Caucasian women [5]. However, two statistics have alarming implications: firstly, that breast cancer occurs more commonly among pre-menopausal women and secondly, that their breast cancer is likely to be detected at a more advanced stage, resulting in poor mortality rates [2, 6].

Late detection may be due to lack of participation in breast cancer screening measures. To ascertain whether this is the case, there has been considerable research effort mostly in the USA [7–10] but also in other countries such as the United Kingdom, into the breast cancer screening behaviours of immigrant African women. [11, 12]. International studies indicate that as in the case of immigrant women from other minority ethnic groups [13–16] culturally-based beliefs about cancer have an important impact on immigrant African women’s cancer screening behaviours [8, 17, 18]. For example, fatalistic attitudes are particularly prominent in African cultures [19]. Studies conducted in the USA [9, 20] and the UK [12] demonstrate that fatalism among immigrant African women results from a combination of fear and avoidance. Breast cancer, or any form of cancer for that matter, is seen as an inescapable death sentence and that early detection by means of screening will make no difference to that outcome. Moreover, like Chinese immigrant women [15, 21], most women from Africa refuse to think about cancer when they are asymptomatic. In addition it has been suggested in the study conducted by Ndukwe and colleagues [22], that cancer carries a stigma and therefore is a taboo subject or is only discussed in strict confidence [23].

African immigrants may have different model of health care that influences their ideas about illness and health seeking behaviours [10, 17]. Many African seek medical advice only when they are symptomatic. International studies indicate that acceptance of the concept of screening as a secondary preventive measure, which is well established in many Western countries, may be foreign to women from minority cultures. In the absence or signs or symptoms of cancer, immigrant women perceive no need for breast screening measures [10, 24–26].

Studies have also demonstrated that African immigrant women often have little or no knowledge about breast cancer screening measures [18, 27]. This is not surprising since early detection of breast cancer is not seen as a priority in many African countries where most health outlays are devoted to HIV alleviation [2]. In the only published Australian study, conducted by Oguniji and colleagues [28], West African immigrant women reported being unaware of breast cancer prior to migrating to Australia.

As in the case of other minority groups, international studies have also identified common barriers to mammographic screening such as lack of transport, lack of English proficiency, the likely costs in the absence of health insurance and fear of cancer diagnosis [10, 13, 24, 25, 29, 30]. To this list must be added ignorance of the existence of screening measures; making it quite likely that many African Australian women may not aware of the national screening program which offers women aged between 50 and 74 free mammograms every two years [31]. However, there is no definite proof of this since studies about breast cancer screening behaviours among African Australian women are scarce to non-existent.

This situation leads us to assert that a culturally sensitive instrument to assess African Australian women’s knowledge of and attitudes towards early breast cancer detection measures is essential. The aim of the present study was to assess and report the psychometric properties of the Breast Cancer Screening Beliefs Questionnaire [BCSBQ] among African Australian women. Recently, the BCSBQ has been validated among Arabic [32], Korean [33] and Indian [34] communities. All these studies have demonstrated a high degree of reliability, suggesting that women from minority cultures in Australia share certain cultural beliefs about breast cancer and breast cancer screening. However, to date the extent to which these cultural beliefs concur with the views of African Australian women has been unknown. In this study we demonstrate how the BCSBQ has been used to fill this lacuna.

## Methods

A cross sectional study design was used in this study.

### Participants and recruitment

The participants of this study were African Australian women who met the following criteria: (1) being aged 18 years and over; (2) were resident in Sydney and (3) had no history of breast cancer. Women in the last category were excluded because of the possibility that their cancer diagnosis might have changed their beliefs, knowledge and attitudes toward breast cancer and screening behaviours. The term *African Australian women* refers to any female of self-reported African descent who has migrated to Australia.

### Recruitment and data collection

Convenience sampling was utilised in recruiting the participants for the study. Leaders of African community women’s organisations and associations in Sydney and also African churches were approached to gain access to their female members. The second author, who is from African background, provided details of the study to community leaders and explained its aims and procedures to them. While some leaders offered assistance in distributing the questionnaire, some preferred the second author’s personal attendance in meetings at which the questionnaires were distributed, completed and collected. Participants also had the option of returning the questionnaire in a stamped, addressed envelope. Other means of gaining participation included personal networking and attendance at African community events and end-of-the-year activities.

Data were collected between October 2013 and December 2014. Prior to data collection, the participants were notified that ethics approval for the study had been obtained. An information sheet containing detailed information was given to the women and a participant’s completion of the questionnaire was considered to constitute her written consent.

### The BCSBQ instrument

The BCSBQ, first developed in English, is a 13-item instrument designed to investigate behaviours in terms of three subscales as follows: 1) *Attitudes* towards general health check-ups, with a subscale of four items designed to determine the participants’ attitudes to undergoing such checks in the absence of signs and symptoms of disease; 2) *Knowledge and perceptions* about breast cancer, with a subscale of four items designed to elicit information on the participants’ cultural beliefs regarding breast cancer; 3) *Barriers* to mammographic screening practices, with a subscale of five items covering what participants perceive as psychological and practical barriers that prevent or at least discourage them from participating in mammographic screening. For the purpose of this paper, the term “The African-Australian BCSBQ” is used.

**Fig. 1:**
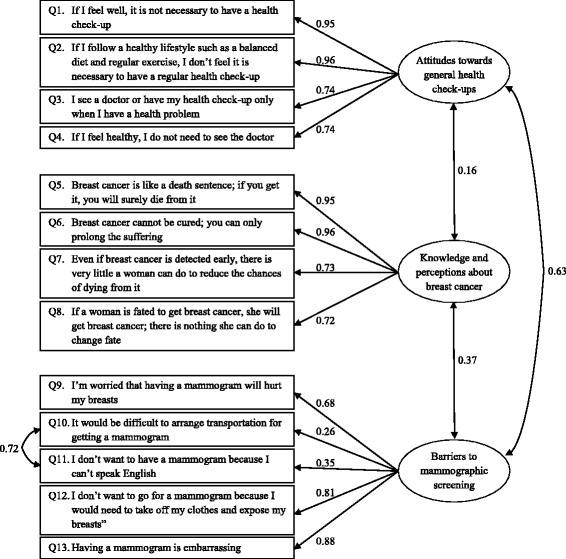
Path diagram of a confirmatory factor analysis of the African Breast Cancer Screening Beliefs Questionnaire. The values correspond to the standardized estimates

Figure 1 gives a brief description of each item of the subscales. Participants were asked to rate these items along a five point Likert scale ranging from ‘Strongly agree’ (score of 1) to ‘Strongly disagree’ (score of 5). Lower scores in each subscale indicated the less proactive attitudes, less knowledge or greatest barriers to mammography. Unlike the BCSBQs administered earlier to linguistically homogenous groups of Chinese, Korean and Arabic women which were translated into their respective languages, the wide linguistic diversity of African languages meant that the African-Australian BCSBQ had to be administered in English. This was not a great disadvantage because as set out below, most participants claimed to have good English fluency. In order to ensure the clarity, understand ability and readability of the instrument, it was piloted among 15 African Australian women with various demographic backgrounds who unanimously confirmed its comprehensibility.

In addition to the information obtained from the African-Australian BSCBQ, demographic information about age, length of stay in Australia, English language proficiency and highest level of educational attainment was collected. The participants were also asked about the frequency with which they undertook the basic screening practices of breast awareness (knowing the normal look and feel of their breasts, without needing to apply a special technique), clinical breast examination (CBE) and mammography.

### Sample size

We planned to recruit over 200 participants to ensure an adequate sample size required for factor analysis of the 13-item BCSBQ, using the rule of thumb of 10 subjects per item [35]. Approximately 500 African Australian women were invited to participate in the study. The questionnaire was returned by 284 women, giving a response rate of 56.8 %. Among the 20 women excluded from the study, 14 had history of breast cancer and six did not complete the questionnaire. The final sample size was thus 264, well within the acceptable sample size for the confirmatory factor analysis (CFA).

### Statistical analysis

The demographic characteristics of the participating women were summarized using descriptive statistics. The originally designed 3-factor structure of the BCSBQ questionnaire was first examined using a CFA of this cohort. The covariance matrix of the 13 items was first computed and then factor-analyzed using maximum likelihood method. Goodness-of-fit of the factor model was assessed by the fit indices with respective common criteria, namely the root mean square error of approximation (RMSEA ≤ 0.06), standardized root mean square residual (SRMR ≤ 0.08), comparative fir index (CFI ≥ 0.95), and non-normed fit index (NNFI ≥ 0.95), which addressed the parsimony correction, absolute fit, and comparative or incremental fit, as recommended [36, 37]. When there were doubts about inadequate fit, addition of covariance between items was made based on the largest modification index [38]. If the 3-factor structure could not be confirmed by the above criteria, an exploratory factor analysis (EFA) would be conducted. The number of factors would be determined by the scree plot. Factor loadings after a varimax rotation would be computed.

The subscale scores of the questionnaire were then calculated on the same basis as the original version previously reported by Kwok et al. [39]. The half-rule was applied, i.e., missing values were imputed by the mean of the responses in the same subscale, provided at least half of the items in that subscale were valid. Floor and ceiling effects were examined to determine whether the 5-point Likert scale was sufficient to distinguish the responses at the two extremes clearly. Internal consistency reliability for each subscale was evaluated by the Cronbach’s alpha. A good Cronbach’s alpha should range from 0.7 to 0.9. A Cronbach’s alpha lower than 0.7 may indicate a low degree of homogeneity whereas a value much higher than 0.9 may imply item redundancy [35].

Construct validity was also examined by testing three hypotheses regarding the association between the subscale scores and the frequency of screening practices and/or education level: (1) those who performed breast awareness exercises and/or presented themselves for CBE and mammograms more frequently, were likely to have a more proactive attitude towards breast cancer screening as reflected by a higher *Attitude* subscale score; (2) those who achieved higher education levels would be more knowledgeable about breast cancer screening and thus record higher *Knowledge* subscale scores; (3) those who had more frequent screening practices were associated with fewer barriers to breast cancer screening, which resulted in a higher *Barriers* subscale score. Because of the ordinal-type nature of the frequency of screening practices and education level, the trend was tested by a Cuzick’s non-parametric test [40]. All statistical analyses were performed using SAS version 9.3.

### Ethical issues

The research project was approved by the Human Ethics Committee of Western Sydney University – number H9759.

## Results

The demographic characteristics of the 264 participants who completed the questionnaire are summarized in Table 1. Their ages ranged from 18 to 69, with a mean (standard deviation) of 49.5 (10.4) years. They had lived in Australia for a mean of 8.7 (4.9) years. Most were married (66.2 %), had tertiary or higher education qualifications (58.3 %), spoke English at home (74.6 %) and rated their English level as good or very good (86.7 %).

**Table 1:** Demographic characteristics of the 264 participants

Characteristic	*N*	(%)
Age (year) (Mean: 49.5, SD: 10.4, missing: *N* = 7)		
>20	1	(0.4)
20 – 29	10	(3.9)
30 – 39	32	(12.5)
40 – 49	77	(30.0)
50 – 59	92	(35.8)
60 – 69	45	(17.5)
Country of birth		
East Africa	17	(6.4)
West Africa	95	(36.0)
North Africa	88	(33.3)
South Africa	64	(24.2)
Language spoken at home		
African	18	(6.8)
Dinka	40	(15.2)
English	197	(74.6)
Others	9	(3.4)
Length of stay in Australia (year)		
(Mean: 8.7, SD: 4.9, missing: *N* = 12)		
0 – 5	70	(27.8)
6 – 10	111	(44.0)
11 – 15	52	(20.6)
16 – 20	12	(4.8)
21 – 25	6	(2.4)
26 or above	1	(0.4)
Marital status (missing: *N* = 1)		
Single	19	(7.2)
Married/defacto (living together)	174	(66.2)
Divorced/separated	60	(22.8)
Widowed	10	(3.8)
Education level		
Primary school	20	(7.6)
Secondary school	47	(17.8)
TAFE/college	43	(16.3)
Tertiary or above	154	(58.3)
Current employment status (missing: *N* = 1)		
Employed, full time	125	(47.5)
Employed, part time	94	(35.7)
Unemployed	38	(14.4)
Retired	6	(2.3)
Self-rated English level		
Little	14	(5.3)
Average	21	(8.0)
Good	79	(29.9)
Very good	150	(56.8)

The CFA of the hypothesized 3-factor structure of the African-Australian BCSBQ resulted in a chi-square statistic = 680.9 (degrees of freedom = 62, *p*-value < 0.001), RMSEA = 0.19 (95 % confidence interval (CI) = 0.18 to 0.21), SRMR = 0.15, CFI = 0.78 and NNFI = 0.72. After examining the modification index, a covariance between Q10 and Q11 was added to the factor model and the fit statistics improved but still did not satisfy the pre-specified criteria: chi-square statistic = 422.1 (degrees of freedom = 61, *p*-value < 0.001), RMSEA = 0.15 (95 % CI = 0.14 to 0.17), SRMR = 0.13, CFI = 0.87 and NNFI = 0.83. The final CFA model is shown in Figure 1.

Since the hypothesized 3-factor structure could not be confirmed through the CFA, an EFA was performed as planned. The eigenvalues for the first five factors were 6.04, 2.21. 1.31, 1.03 and 0.56. Having examined the scree plot, a 4-factor model was identified, the varimax-rotated loadings of the four factors being presented in Table 2. These factors together explained 81.5 % of the total variance. Items having a loading with magnitude ≥ 0.4 within a particular factor were considered to be its major component and are highlighted. All items on the *Attitude* and *Knowledge* subscale were loaded more heavily on the first and second factors respectively, while the items on the *Barriers* subscale loaded the third and fourth factors. As noted earlier, the third factor consisted of three items designed to establish psychological barriers to mammographic screening. These were: “*I’m worried that having a mammogram will hurt my breasts* (Q9),” “*I don’t want to go for a mammogram because I would need to take off my clothes and expose my breasts* (Q12)” and “*Having a mammogram is embarrassing* (Q13)”. The fourth factor consisted of two items related to practical barriers: “*It would be difficult to arrange transportation for getting a mammogram* (Q10)” and “*I don’t want to have a mammogram because I can’t speak English* (Q11)”. Therefore, apart from examining the main *Barrier* scale, we split the remaining validation into two “daughter” subscales, namely *Psychological* and *Practical* barriers.

**Table 2:** Rotated factor loadings of the exploratory factor analysis of the African Breast Cancer Screening Beliefs Questionnaire

	Factor loadings			
Items	Factor 1	Factor 2	Factor 3	Factor 4
Attitudes towards general health check-ups				
Q1	**0.84**	0.31	0.00	0.15
Q2	**0.83**	0.34	0.01	0.17
Q3	**0.83**	0.26	0.03	0.15
Q4	**0.82**	0.30	0.09	0.11
Knowledge and perceptions about breast cancer				
Q5	0.29	**0.82**	0.11	0.25
Q6	0.28	**0.84**	0.16	0.21
Q7	0.35	**0.79**	0.06	0.09
Q8	0.36	**0.79**	0.13	0.00
Barriers to mammographic screening				
Q9	0.13	−0.08	**0.85**	0.08
Q10	0.21	0.13	0.11	**0.91**
Q11	0.17	0.19	0.18	**0.89**
Q12	−0.06	0.18	**0.85**	0.16
Q13	0.03	0.24	**0.86**	0.07

Table 3 presents the distributions of the score of the subscales. Five women did not answer all items which meant that the half-rule could not be applied to compute the score of the *Barriers* subscale and its two daughter subscales. The subscales under *Attitude* and *Knowledge* had a range from 0 to 100, while the *Barrier* subscale had a minimum score of 5. There were very mild (≤3 %) floor and ceiling effects for the three original as well as the *Psychological* barriers subscale, but 15.5 % of the women attained the maximum score of 100 for the *Practical* barrier subscale. The Cronbach’s alpha of the *Attitude, Knowledg*e and *Barriers* subscales were 0.92, 0.91 and 0.77 respectively. However, after splitting the *Barriers* subscale into two, the Cronbach’s alphas improved to 0.84 for *Psychological* barriers and 0.86 for *Practical* barriers.

**Table 3:** Distribution of the subscale scores of the 13-item African Breast Cancer Screening Beliefs questionnaire and Cronbach’s Alpha

Subscale	*N*	Mean	Standard deviation	Median	% at floor	% at ceiling	Cronbach’s alpha
Attitudes towards general health check-ups	264	41.4	25.8	25.0	3.0	2.7	0.92
Knowledge and perceptions about breast cancer	264	44.7	26.2	37.5	1.5	1.9	0.91
Barriers to mammographic screening	261	56.7	16.7	55.0	0	1.1	0.77
Psychological barriers	259	44.3	22.2	41.7	0.4	1.5	0.84
Practical barriers	261	74.7	17.2	75.0	0	15.5	0.86

The mean scores of the three original subscales and two daughter subscales, stratified by the participants’ education levels and frequency of screening practices, are shown in Table 4. Women with higher education levels obtained significantly higher scores in all subscales (all *p*-values < 0.01). The *Attitude* subscale score was significantly higher among those who had more frequent CBEs and mammograms, but marginally insignificantly higher among those who performed more frequent breast awareness exercises (*p*-value = 0.068). The main *Barriers* subscale score in contrast, was significantly associated only with the frequency of breast awareness exercises (*p*-value = 0.028) and not with frequency of CBEs (*p*-value = 0.104) and mammography (*p*-value = 0.451). However, after splitting this factor into two daughter subscales, the *Practical* barrier score showed itself to be significantly associated with all three screening practices (all *p*-values < 0.05).

**Table 4:** Construct validity of the African Breast Cancer Screening Beliefs Questionnaire

		Attitudes towards general health check-ups	Knowledge and perceptions about breast cancer	Barriers to mammographic screening		
				Original subscale	Psychological barriers	Practical barriers
	*N* (%)	Mean (SD)	Mean (SD)	Mean (SD)	Mean (SD)	Mean (SD)
Education level						
Primary school	20 (7.6)	24.1 (15.9)	25.0 (16.8)	40.3 (17.1)	33.8 (16.3)	50.0 (24.7)
Secondary school	47 (17.8)	26.9 (19.3)	26.7 (18.6)	51.5 (13.4)	39.9 (16.1)	68.9 (16.5)
TAFE/college	43 (16.3)	40.1 (22.8)	39.8 (22.8)	55.1 (16.3)	40.4 (21.0)	75.6 (18.5)
Tertiary or above	154 (58.3)	48.5 (26.5)	54.1 (25.6)	60.8 (15.9)	48.1 (24.0)	79.5 (12.0)
*P*-value for trend		<0.001	<0.001	<0.001	0.003	<0.001
Breast awareness						
At least once a month	23 (8.7)	42.7 (33.6)	44.3 (30.3)	63.5 (19.0)	52.2 (26.3)	80.4 (15.5)
Once every few months	92 (34.8)	42.7 (24.9)	47.2 (28.4)	56.2 (15.2)	40.5 (22.0)	79.1 (10.7)
Once a year	30 (15.2)	53.8 (23.6)	57.3 (21.3)	61.9 (14.1)	50.0 (23.3)	79.7 (10.5)
Never	109 (41.3)	35.6 (24.0)	38.0 (23.2)	53.6 (17.6)	43.8 (20.3)	67.9 (21.5)
*P*-value for trend		0.068	0.032	0.028	0.748	<0.001
Clinical breast examination						
A year or less	4 (1.5)	71.9 (24.2)	64.6 (12.5)	72.5 (19.4)	66.7 (28.1)	81.3 (16.1)
More than a year and less than two years	16 (6.1)	35.2 (24.2)	52.0 (28.6)	61.3 (21.2)	47.4 (29.0)	82.0 (11.2)
Two to three years	27 (10.2)	52.1 (24.4)	53.9 (23.8)	61.1 (13.7)	50.3 (22.5)	77.3 (8.5)
More than three years	27 (10.2)	51.9 (25.6)	54.2 (26.9)	53.7 (14.9)	38.3 (19.2)	76.9 (17.9)
Never had one	190 (72.0)	38.3 (25.2)	41.0 (25.7)	55.7 (16.7)	43.6 (21.5)	73.3 (18.3)
*P*-value for trend		0.020	0.004	0.104	0.203	0.045
Mammogram						
Once a year	10 (3.8)	58.8 (27.0)	53.1 (26.4)	59.0 (14.1)	37.5 (23.0)	91.3 (11.9)
Once every two years	113 (42.8)	42.1 (25.3)	42.2 (25.6)	57.4 (14.1)	43.6 (20.7)	78.1 (12.3)
Once every three years or more	45 (17.0)	46.9 (27.2)	52.5 (26.8)	53.0 (16.1)	38.5 (20.4)	74.7 (18.9)
Never had one	96 (36.4)	36.3 (24.6)	43.1 (26.2)	57.3 (19.8)	48.9 (24.0)	68.8 (19.7)
*P*-value for trend		0.041	0.975	0.451	0.412	<0.001

*Abbreviation*: *SD* standard deviation

## Discussion

Increasing cancer screening rates among minority populations is vital because early detection is central to reducing morbidity and mortality. This fact lends particular importance to our BCSBQ-based study because it constitutes the first step towards investigating breast cancer screening behaviour and practices among the fast-growing population of African immigrants to Australia.

In contrast to the BCSBQ studies conducted among Korean, Indian and Arabic immigrant populations in which the results supported the original 3-factor model, the EFA results of the present study demonstrated that the African-Australian BCSBQ could be conceptualized as a 4-factor model. The third subscale, viz, *Barriers* to mammography, needed to be split into two factors namely *Psychological* and *Practical* barriers. The three items on the *Psychological* (Q9, Q12, Q13) subscale proved to be of little help in evaluating the participants’ thinking on this issue and in fact masked the effect of the two items on the *Practical* barriers (Q10, Q11) subscale. This became clear after the three *Psychological* items were effectively removed from the main *Barriers* factor which had revealed that the Cronbach’s alpha improved and the three hypothesis tests became (more) significant. *Practical* barriers such as transportation and English proficiency, had a greater impact on African Australian women’s screening behaviours than the *Psychological* barriers set out above. This finding is in agreement with those of overseas studies conducted among African immigrant women [10, 24, 30]. Despite the fact that the majority of this cohort had good or very good English proficiency, the *practical* barriers subscale does include an item relating to concerns about English. It may be that language concerns occur within the technical and medical context of mammograms, and/or that women of African ancestry find it difficult to discuss their modesty concerns. Our findings may indicate that in the future use of this instrument for African participants, the three main *Psychological* barrier items can be regarded as a separate factor that is separate from the *Practical* barrier items. This is in line with a similar modification of the BCSBQ conducted among Hong Kong Chinese in which the two *Practical* barrier items were not included because they had proved to be irrelevant [41].

Similar to the original and other versions, the internal consistency of the three subscales proved excellent, with Cronbach’s alpha ranging from 0.77 to 0.92, comfortably above the acceptable level of 0.70 recommended by Hair et al. [42]. After splitting the *Barrier subscale* into two, the Cronbach’s alpha improved to 0.86 for *Practical* barriers. However, one drawback of the division of the main *Barrier* factor was the inflation in the ceiling effect which increased from 1.1 % in the original to 15.5 % under the new the Practical factor. This was not surprising since one of the two items related to concerns about English and more than half the African Australian women in this cohort had attained tertiary or higher education qualifications, spoke English at home and rated their English level as very good. Although the subscale score could not be distinguished among this proportion of participants, it had better reliability and discriminative power than the original main *Barrier* factor across the whole spectrum as evidenced by the Cronbach’s alpha and the three hypothesis tests. This again supported the separation of the main *Barrier* subscale into two daughter factors.

The African-Australian BCSBQ also demonstrated good construct validity with the associations between the three subscales and the frequency of screening practices. In line with studies focused on Chinese, Arabic, Korean and Indian immigrant women, the Attitudes subscale showed significant associations with both CBE and mammographic screening. Our findings on this score are supported by the claim that immigrant women often have different concepts of preventive care and that having cancer screening while asymptomatic is foreign to their health practices [24, 26, 43, 44]. It is evident that the *Practical* barrier subscale was more significantly associated with breast cancer screening practices, rather than *Psychological* barriers. This is consistent with the findings of overseas studies which have demonstrated that transportation and language appear as key barriers to mammographic screening among African immigrant women [10, 29].

Our findings also demonstrate that as in the validation study conducted among Korean Australian women [33], the education level of the African group was significantly associated with the three subscales This may be explained by the fact that greater levels of educational attainment create more positive responses to having health check-ups, improving knowledge about breast cancer and lessening the impact of barriers to mammographic screening. This is evident from our finding that African Australian women with better education levels have better understandings of preventative measures, something which counteracts traditional health beliefs focused on curative aspects. Overseas studies across cultural groups likewise indicate education levels as a predictor of women’s screening behaviours [18, 45, 46].

The limitations of this study should be noted when applying the findings. Firstly, participants were born in African countries but resided in Australia. Therefore, these results may not be generalizable to African women living in their home countries. Secondly the study utilised self-reported measures of breast cancer screening practices that could have been over or under-reported. Further studies with adequate verification of self-reported information built into their design, are warranted.

## Conclusion

The psychometric properties assessment of the African-Australian BCSBQ reported in this paper confirm that the instrument is a valid and reliable tool for assessing breast cancer beliefs and attitudes towards screening practices among African-Australian women.
